# An Atherogenic Paigen-Diet Aggravates Nephropathy in Type 2 Diabetic OLETF Rats

**DOI:** 10.1371/journal.pone.0143979

**Published:** 2015-11-25

**Authors:** Masanori Nozako, Takashi Koyama, Chifumi Nagano, Makoto Sato, Satoshi Matsumoto, Kiminobu Mitani, Reiko Yasufuku, Masayuki Kohashi, Tomohiro Yoshikawa

**Affiliations:** 1 Free Radical Research Project, Otsuka Pharmaceutical Co., Ltd., Tokushima, Tokushima, Japan; 2 Department of Toxicology, Drug Safety Research Center, Tokushima Research Institute, Otsuka Pharmaceutical Co., Ltd., Tokushima, Tokushima, Japan; University of Louisville, UNITED STATES

## Abstract

Diabetic nephropathy develops in association with hyperglycemia, is aggravated by atherogenic factors such as dyslipidemia, and is sometimes initiated before obvious hyperglycemia is seen. However, the precise mechanisms of progression are still unclear. In this study, we investigated the influence of an atherogenic Paigen diet (PD) on the progression of nephropathy in spontaneous type 2 diabetic OLETF rats. Feeding PD to male OLETF rats for 12 weeks caused an extensive increase in excretion of urinary albumin and markers of tubular injury such as KIM-1 and L-FABP, accompanied by mesangial expansion and tubular atrophy. PD significantly increased plasma total cholesterol concentration, which correlates well with increases in urine albumin excretion and mesangial expansion. Conversely, PD did not change plasma glucose and free fatty acid concentrations. PD enhanced renal levels of mRNA for inflammatory molecules such as KIM-1, MCP-1, TLR4 and TNF-α and promoted macrophage infiltration and lipid accumulation in the tubulointerstitium and glomeruli in OLETF rats. Intriguingly, PD had little effect on urine albumin excretion and renal morphology in normal control LETO rats. This model may be useful in studying the complex mechanisms that aggravate diabetic nephropathy in an atherogenic environment.

## Introduction

Diabetic nephropathy (DN) is one of the most important microvascular complications and the major determinant of morbidity and mortality in diabetic patients. Although chronic hyperglycemia is a major cause of DN, other factors such as obesity, dyslipidemia and hypertension have also been implicated in the development of DN, where genetic and environmental factors are responsible for triggering a complex series of pathophysiological events [1, 2], and initiation sometimes occurs before overt hyperglycemia is seen.

Abnormal metabolism of glucose or free fatty acids (FFAs) via mitochondrial pathways and activation of nicotinamide adenine dinucleotide phosphate (NADPH) oxidases via protein kinase C (PKC) have been recognized as contributors to the production of reactive oxygen species, which would lead to the development of DN, accompanied by glomerular hyperfiltration, proteinuria, glomerular and interstitial fibrosis [2, 3]. In addition, inflammatory cytokines such as tumor necrosis factor-α (TNF-α), chemokines including monocyte chemoattractant protein-1 (MCP-1/CCL2) and vascular cell adhesion molecule-1 (VCAM-1) have been shown to contribute to the development of DN, where activation of nuclear factor κB (NF-κB) signaling, the classic inflammation pathway, is a key mechanism that regulates inflammatory cytokines in a complex manner [2, 4].

Hyperglycemia, FFAs and obesity may promote oxidative stress and activate NF-κB through PKC to rapidly stimulate the expression of cytokines [2, 3, 5, 6], and hypercholesterolemia is reported to be a risk factor for nephropathy in patients with type 1 [7, 8] and type 2 [9, 10] diabetes. Intriguingly, these mechanisms resemble those responsible for the progression of macrovascular complications such as atherosclerosis [11–13]. Therefore, atherogenic factors that induce dyslipidemia, enhanced oxidative stress and inflammation could also affect the progression of DN. However, the effect of atherogenic factors on the progression of DN varies depending on the type of diabetes and differences in genetic background. Consequently, the mechanisms of the progression of DN in an atherogenic environment have not been fully elucidated.

The Otsuka Long Evans Tokushima Fatty (OLETF) rat is a model of type 2 diabetes mellitus with accompanying obesity [14], hyperlipidemia and diabetic complications such as nephropathy [15]. Nephropathy progress slowly in OLETF rats; significant pathological changes are observed in the kidney after the age of 40 weeks, including extracellular matrix accumulation in the mesangium, thickening of the glomerular basement membrane and glomerulosclerosis, resembling human diabetic glomerulopathy [15]. A high-cholesterol diet formulated by Paigen et al., the “Paigen diet (PD)” has been used widely to induce atherosclerosis in experimental animals [16, 17]. The diet is reported to aggravate nephropathy in streptozotocin-induced diabetic rats [18], but there have been no reports on nephropathy in type 2 diabetes.

The present study was performed to elucidate the effect of the atherogenic PD on the progression of kidney injury in OLETF rats with type 2 diabetes. In this study, we clearly showed that kidney impairment in OLETF rats abruptly deteriorates following feeding with the PD, accompanied by severe mesangial expansion and tubular atrophy. Intriguingly, PD caused negligible damage to the kidney in normal Long Evans Tokushima Otsuka (LETO) rats.

## Materials and Methods

### Ethics Statement

This study was performed in strict accordance with the Guidelines for the Animal Care and Use of Otsuka Pharmaceutical Co., Ltd. which conforms to the international norms stipulated by the Ministry of Health, Labour and Welfare, Japan. The study protocol was approved by the Institutional Animal Care and Use Committee of the Otsuka Pharmaceutical Co., Ltd. (Permit Numbers: 13–0240, 14–0285). Every endeavor was made to maximize the welfare of the animals and minimize their suffering as follows: Animals were treated carefully by experts who had undergone education and training on how to maintain a healthy environment and reduce distress. Measurements taken in the absence of anesthesia were minimized to reduce undue stress, and animals were euthanized by bleeding under ether or isoflurane anesthesia.

### Animals

Male OLETF and LETO rats (specific pathogen-free, 4–7 weeks old) were obtained by in-house breeding or purchased from Hoshino Laboratory Animals, Inc. (Ibaraki, Japan). The rats were acclimatized and housed in individual plastic cages in animal rooms maintained at 23°C ± 2°C and 60% ± 10% humidity with 12-h cycles of light and dark. They fed freely on normal chow (NC), Western diet (WD) or PD. PD includes 1.25% cholesterol, 0.5% sodium cholate and 15% cocoa butter; WD includes 0.125% cholesterol and 20% lard. The diets were prepared by Oriental Yeast Co., Ltd. (Osaka, Japan). As OLETF rats are susceptible to artificial stresses encountered during some kinds of measurements, the rats were treated carefully, and some parameters were measured separately using different rats to reduce such artificial influences.

### Experimental design

We first examined the effects of different atherogenic diets (PD and WD) on the nephropathy of OLETF rats using normal control LETO rats as reference in Experiment 1. Then, we examined on the gradients of PD affecting the nephropathy in Experiment 2, and the mechanism of the deterioration induced by PD in Experiment 3.

#### Experiment 1

Nineteen 5-6-week-old OLETF rats were randomly allocated to three groups consisted as follows: NC and WD group (five rats each), and PD group (nine rats). Ten 5-6-week-old LETO rats were randomly allocated to two groups (NC and PD group, five rats each). These rats fed on each diet freely for 12 weeks, and were then euthanized for histological analysis.

#### Experiment 2

Thirty-two OLETF rats aged 6 weeks were randomly allocated to four groups (eight rats each) consisted as follows: 1) NC group, 2) CA diet (PD diet without 1.25% cholesterol and cocoa butter) group (CA group), 3) High-fat diet (PD diet without 0.5% CA) group (HF group) and 4) PD group. These rats fed on each diet freely for 5 weeks, and were then euthanized for histological analysis.

#### Experiment 3

Thirty-one OLETF rats aged 6 weeks were randomly allocated to two groups as follows: NC group (eight rats) and PD group (23 rats). Five LETO rats served as normal control groups. These rats fed on each diet freely for 12 weeks, and were then euthanized for histological, immunohistochemical and gene expression analysis. Blood pressures were measured using another set of rats constituted as follows: OLETF rats fed NC group, OLETF rats fed PD groups and LETO rats fed NC groups (eight rats each). These rats fed on each diet freely for 12 weeks, and were then euthanized.

### Blood, urine and tissue collection

Blood was collected from the tail vein without anesthesia. Urine was collected for 16 hours in a metabolic cage. Rats were operated on at 5 or 12 weeks after the onset of feeding of each diet, under ether or isoflurane anesthesia. After celiotomy, the kidneys were isolated, weighed, and fixed in 4% paraformaldehyde solution (Wako Pure Chemical Co. Ltd., Osaka, Japan). Part of each kidney was embedded in OCT compound and frozen in cold acetone.

### Measurement of urine and plasma markers

Glucose, total cholesterol (TC), non-esterified fatty acid, urea nitrogen and creatinine were measured using reagents provided by Wako Pure Chemical Co., Ltd. (Osaka, Japan). N-acetyl-β-D-glucosaminidase (NAG) and triglyceride (TG) were measured using reagents from Shionogi & Co., Ltd. (Osaka, Japan) and Toyobo Co., Ltd. (Osaka, Japan), respectively. Albumin and insulin were measured using Rat Albumin and Insulin ELISA kits (Shibayagi Co., Ltd., Gunma, Japan), respectively. liver fatty-acid-binding protein (L-FABP), cystatin C and kidney injury molecule-1 (KIM-1) were measured using Mouse/Rat FABP1/L-FABP, Mouse/Rat Cystatin C and Rat TIM-1/KIM-1/HAVCR Immunoassay kits (R&D Systems, Inc., MN, USA), respectively. We measured 15-isoprostane F2t and 8-hydroxy-2'-deoxyguanosine using an 8-Isoprostane EIA kit (Cayman Chemical Company, MI, USA) and Rat New 8-OHdG check ELISA kit (Japan Institute for the Control of Aging, Nikken SEIL Co., Ltd., Shizuoka, Japan), respectively. Creatinine clearance was calculated from the plasma creatinine concentration and urinary creatinine excretion over 16 hours. Plasma lipoprotein profiles were analyzed by high performance liquid chromatography (Tosoh Co., Tokyo, Japan) using the pooled plasma of each group as described previously [19].

### Histological analysis

For the following histological analysis, kidney tissue was embedded in paraffin, sectioned and stained with periodic acid schiff (PAS). For morphometric comparison of glomerular size, the areas of 50 glomeruli in each mouse were measured using image analysis software (WinROOF version 5.8.0; Mitani Corporation, Tokyo, Japan), and the radius was derived from the measured area; volume was calculated by cubing the radius and further multiplying by 4/3π. For semi-quantitative histopathological analysis of glomeruli, 100 glomeruli were examined randomly in each animal. The degree of glomerular sclerosis evaluated on PAS staining was scored from 0 to 4 depending on the relative size of PAS-positive areas such as expanded mesangial areas as follows: 0, 0%-10%; +1, 10%-25%; +2, 25%-50%; +3, 50%-75%; +4, 75%-100%. The analyses were performed by two independent observers in a blinded fashion. Frozen sections were stained with oil red O stain for the analysis of lipid accumulation.

Immunohistochemical analysis was performed using antibodies against CD68 (MCA341GA, Serotec Co., Ltd., Sapporo, Japan), NADPH oxidase 2 (NOX2/Gp91phox, 611415, BD Transduction Laboratories, Tokyo, Japan), KIM-1 (AF3689, R&D Systems, Inc., MN, USA), osteopontin (18621, Immuno-Biological Laboratories Co., Ltd., Gunma, Japan), myeloid-related protein 8 (MRP8, ORB100060, Biorbyt LLC, CA, USA), Toll-like receptor 4 (TLR4, ab22048, Abcam plc, Tokyo, Japan) and tumor necrosis factor-α (TNF-α, ab66579, Abcam plc, Tokyo, Japan). Working dilutions of these antibodies were 1:100, 1:100, 1:200, 1:50, 1:100, 1:500, and 1:100, respectively.

### Gene expression analysis

Total RNA was extracted from renal cortical tissues and purified using an RNeasy Mini kit (Qiagen K.K., Tokyo, Japan). Reverse transcription was performed for 15 min at 42°C and 3 min at 95°C using QuantiTect Reverse Transcription Kit (Qiagen K.K., Tokyo, Japan). Quantitative gene expression was performed with ABI 7500 Real Time PCR System (Applied Biosystems, Tokyo, Japan) using SYBR Green technology. Primers were purchased from Qiagen K.K. (Tokyo, Japan). The product codes of the primers are indicated in S1 Table. Real-time PCR was performed for 40 cycles, consisting of denaturation for 10 sec at 95°C and annealing with extension for 30 sec at 60°C. At the end of the 40 cycles, samples were heated to 95°C to verify that a single PCR product was obtained during amplification. The ratio of each gene and the glyceraldehyde 3-phosphate dehydrogenase (GAPDH) level (relative gene expression number) were calculated by subtracting the threshold cycle number (Ct) of the target gene from that of GAPDH and raising 2 to the power of this difference.

### Measurement of blood pressure

Systolic blood pressure was measured using Non-Preheating Non-Invasive Blood Pressure Monitor for Mice and Rats (MK-2000ST, Muromachi Kikai Co., Ltd., Tokyo, Japan) without anesthesia.

### Statistical analysis

Data are presented as the mean ± SD. The differences between two groups and among more than two groups were analyzed using an unpaired *t*-test and Dunnett’s test, respectively. Synergistic effects were evaluated with the p value of the interaction effect analyzed by two-way analysis of variance (ANOVA). The differences were considered significant at 5% in the two-tailed test. To determine the relationship between plasma cholesterol and urinary albumin excretion (UAE) or PAS score, or between UAE and urinary excretion of tubular injury markers, a Pearson or Spearman correlation coefficient and p value were calculated and a test of no correlation was performed. SAS software (Release 9.1; SAS Institute Inc., Tokyo, Japan) was used for all statistical analyses.

## Results

### Effects of an atherogenic diet on nephropathy in OLETF rats

**Fig 1:**
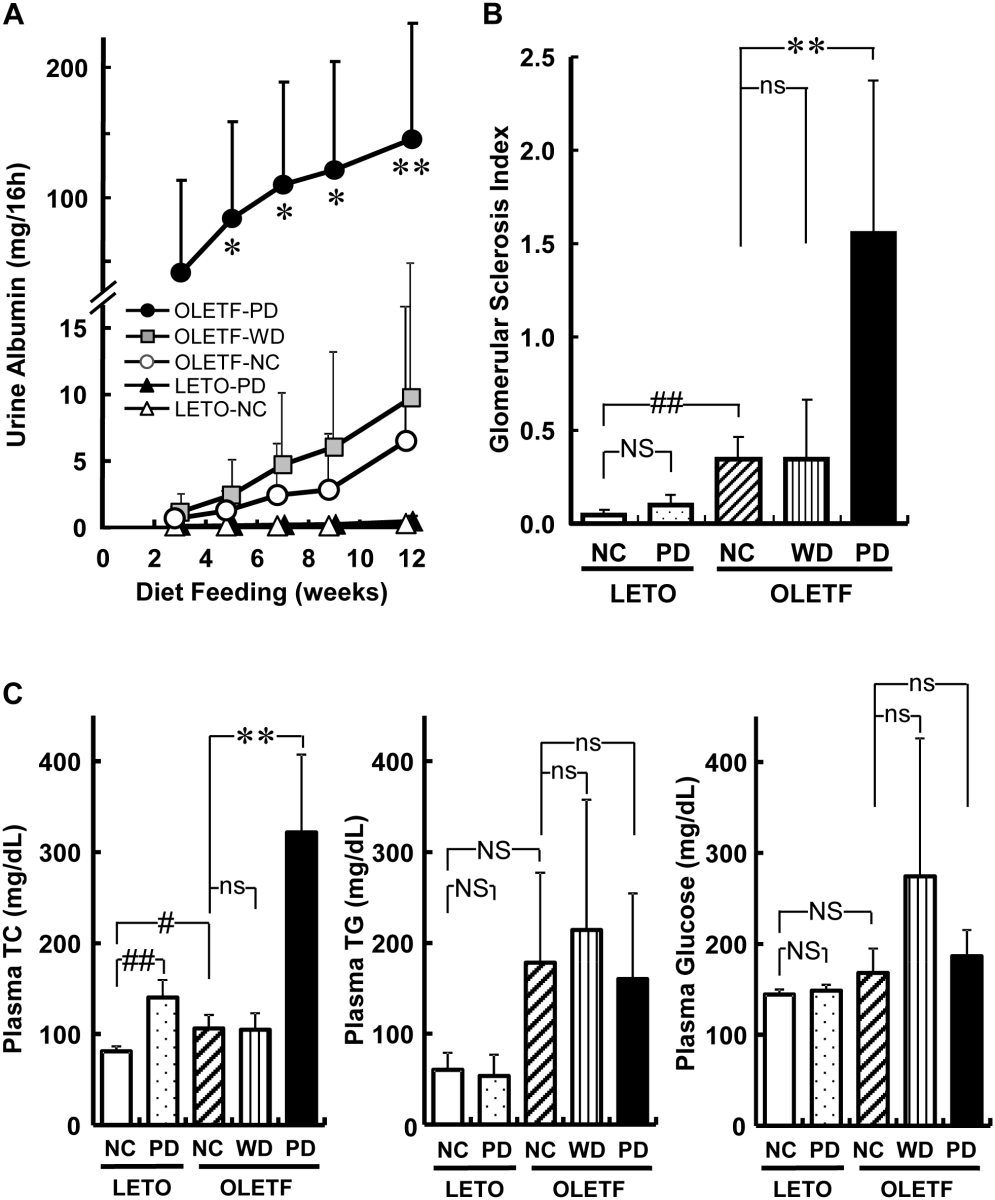
An atherogenic Paigen-diet worsens the nephropathy of OLETF rats. Time-dependent changes in urine albumin excretion (A), semi-quantitative analyses of glomerular sclerosis (B) and plasma lipids and glucose concentrations (C) 12 weeks after starting to feed chow or an atherogenic diet, presented as mean ± SD. There were five animals in the following groups: LETO rats fed normal chow (LETO-NC) and Paigen diet (LETO-PD), and OLETF rats fed NC (OLETF-NC) and Western diet (OLETF-WD); and nine in the OLETF rats fed PD (OLETF-PD) groups. *p < 0.05; **p < 0.01; ns, not significant vs OLETF-NC group among three OLETF groups by Dunnett’s test. #p < 0.05; ##p < 0.01; NS, not significant by unpaired t-test. There were no significant differences between LETO-NC and LETO-PD or LETO-NC and OLETF-NC groups in urine albumin (unpaired t-test). TC, total cholesterol; TG, triacylglycerol.

UAE in OLETF rats fed NC increased gradually with age, while in normal control LETO rats fed NC, UAE remained constant at a very low level (Fig 1A), as reported previously [15]. PD markedly enhanced the UAE in OLETF rats (Fig 1A), and the degree was more rapid and severe than that seen in OLETF rats fed a 40% protein diet [15]. Pathological examination revealed that PD expanded mesangial areas in glomeruli, on evaluation of PAS-positive areas in OLETF rats (Fig 1B). Conversely, PD did not affect UAE or mesangial areas in LETO rats (Fig 1A and 1B). WD, another representative atherogenic diet, increased UAE of OLETF rats, but the increment was much smaller than that seen in OLETF rats fed PD (Fig 1A), and glomerular mesangial areas were little changed by WD (Fig 1B). Plasma creatinine and urea nitrogen concentrations and urine creatinine excretion were similar among the groups (Table 1).

**Table 1:** Metabolic Parameters of LETO and OLETF rats 12 weeks after starting to feed normal chow or atherogenic diets in Experiment 1.

	LETO-NC	LETO-Paigen	OLETF-NC	OLETF-Western	OLETF-Paigen
BW (g)	431 ± 29	475 ± 32	588 ± 107#	655 ± 120	656 ± 88
KW (g)	2.0 ± 0.2	2.1 ± 0.2	2.9 ± 0.6#	3.2 ± 0.7	3.5 ± 0.7
KW/BW (%)	0.47 ± 0.02	0.44 ± 0.02#	0.50 ± 0.03	0.48 ± 0.03	0.53 ± 0.06
FI (g/day)	20 ± 1	20 ± 1	30 ± 8#	30 ± 6	33 ± 6
WI (g/day)	30 ± 3	29 ± 2	38 ± 7#	41 ± 10	41 ± 6
pIns (ng/mL)	3.6 ± 0.9	3.0 ± 1.1	9.5 ± 6.0	10.9 ± 6.0	7.0 ± 3.7
pUN (mg/dL)	19 ± 3	18 ± 2	20 ± 1	17 ± 6	24 ± 10
pCre (mg/dL)	0.85 ± 0.08	0.98 ± 0.24	0.90 ± 0.05	1.24 ± 0.37	1.39 ± 0.85
CCr (mL/min)	1.68 ± 0.23	1.64 ± 0.33	1.77 ± 0.29	1.26 ± 0.37	1.62 ± 0.77
uCre (mg/16 h)	12.2 ± 3.1	14.8 ± 0.9	15.3 ± 2.1	14.1 ± 1.8	17.1 ± 2.9
uGlu (mg/16 h)	2.7 ± 0.2	2.8 ± 0.5	7.0 ± 8.0	11.1 ± 12.0	3.5 ± 1.2
u8IsoP (ng/16 h)	101 ± 17	106 ± 35	187 ± 86	166 ± 58	226 ± 75
u8OHdG (ng/16 h)	223 ± 32	211 ± 44	297 ± 123	233 ± 50	328 ± 151
UV (mL/16 h)	16 ± 7	13 ± 3	16 ± 3	14 ± 6	15 ± 4
n	5	5	5	5	9

Values obtained 12 weeks after starting to feed each diet are presented as mean ± SD.

^#^p < 0.05 compared with LETO-NC group by unpaired t-test. There were no significant differences among the three OLETF groups (Dunnett’s test). BW, body weight; CCr, creatinine clearance; FI, food intake; KW, kidney weight; NC, normal chow; pCre, plasma creatinine concentration; pIns, plasma insulin concentration; pUN, plasma urea nitrogen concentration; uCre, urine creatinine excretion; uGlu, urine glucose excretion; u8IsoP, urine 8-iso-plostaglandine F2α excretion; u8OHdG, urine 8- hydroxydeoxyguanosine excretion; UV, urinary volume WI, water intake.

Plasma TC concentration in OLETF rats fed PD was significantly higher than in OLETF rats fed NC, while plasma glucose and TG concentrations tended to be higher in OLETF rats fed WD than those in OLETF rats fed NC or PD (Fig 1C). Fasting plasma insulin and TG concentrations and HOMA-IR, an index of insulin resistance, in OLETF rats fed PD were lower than in OLETF rats fed NC (Table 2). Food intake, water intake and body weight in OLETF rats were higher than those in LETO rats, but atherogenic diets did not affect these parameters (Table 1). Systolic blood pressure in OLETF rats fed PD for 12 weeks (129 ± 4 mmHg, mean ± SD) was not different from that in OLETF rats fed ND (126 ± 6 mmHg), although these levels were significantly higher than those in LETO rats fed ND (114 ± 4 mmHg).

**Table 2:** Plasma parameters of LETO and OLETF rats after an overnight fast in Experiment 3 (study on blood pressure).

	LETO-NC	OLETF-NC	OLETF-Paigen
TC (mg/dL)	105±14##	146±9	266±44##
TG (mg/dL)	52±22##	220±62	106±39##
NEFA (mEq/L)	1.29±0.20##	0.93±0.19	0.82±0.18
Glucose (mg/dL)	110±6##	141±12	132±9
Insulin (ng/mL)	2.3±0.2##	5.3±0.9	3.1±0.8##
HOMA-IR	16.6±2.0##	48.0±11.0	26.3±7.6##

Values of plasma parameters obtained after an overnight fast 12 weeks after starting to feed each diet are presented as mean ± SD. n = 8.

^##^p<0.01 compared with OLETF-NC by unpaired t-test. HOMA-IR, homeostasis model assessment-Insulin Resistance; NC, normal chow; NEFA, nonesterified fatty acid; TC, total cholesterol; TG, triglyceride.

### High-fat and cholate in Paigen-diet synergistically aggravated glomerular injury in OLETF rats

Next, we tried to determine which ingredient of PD aggravates the nephropathy in OLETF rats by feeding NC, PD (includes 1.25% cholesterol, 0.5% sodium cholate and 15% cocoa butter), PD without sodium cholate (HF-diet), or PD without high fat levels (without 1.25% cholesterol and 15% cocoa butter; CA-diet) to the rats and comparing the UAE and kidney morphology among these rats.

**Fig 2:**
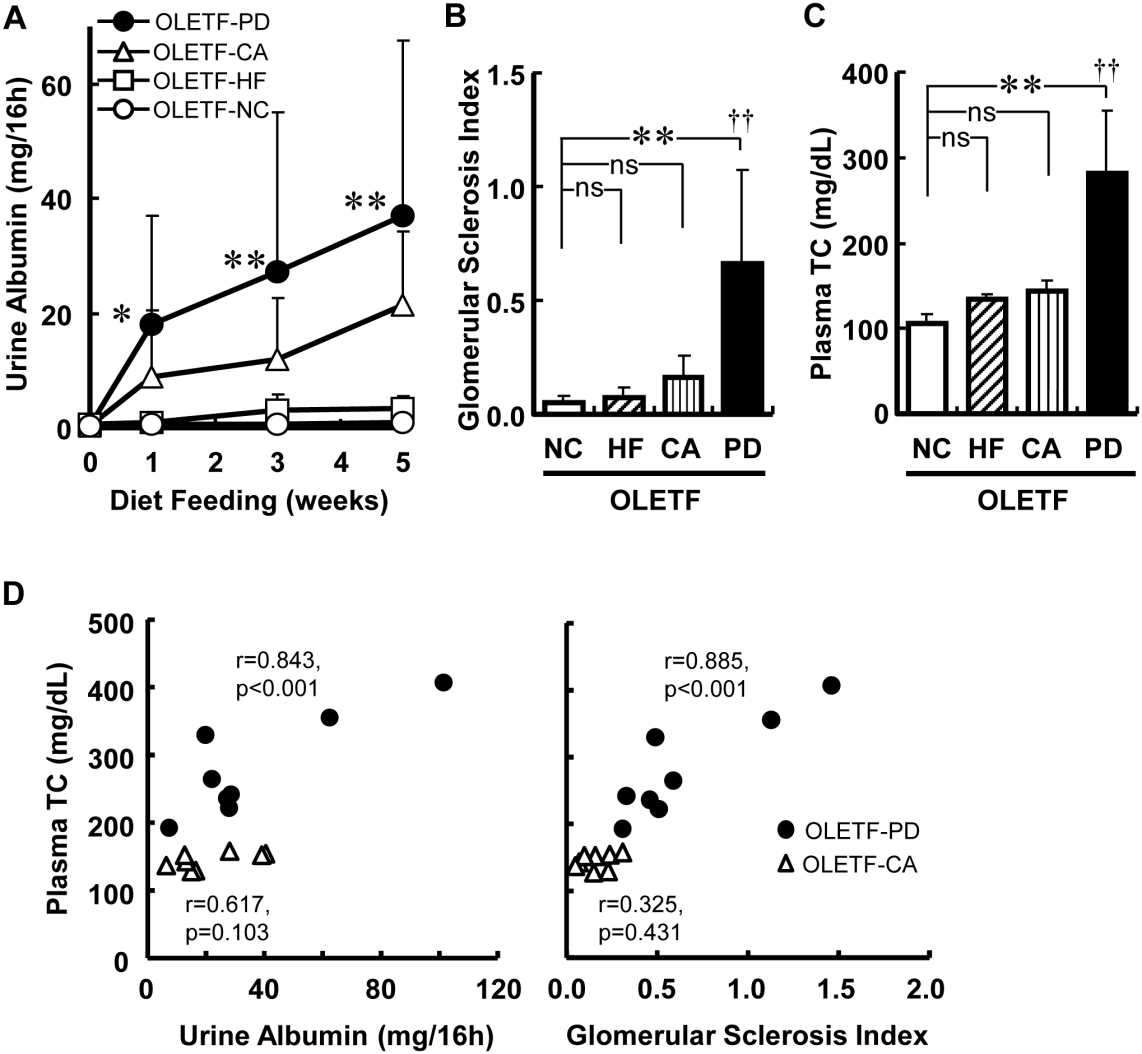
High-fat and cholate in a Paigen diet synergistically worsen the nephropathy of OLETF rats. Time-dependent changes in urine albumin excretion (A), glomerular sclerosis index (B) and plasma total cholesterol (TC) concentration (C) 5 weeks after starting to feed each diet are presented as mean ± SD (n = 8). Relationships between plasma TC concentration and urine albumin secretion or glomerular sclerosis index are shown in D. *p < 0.05; **p < 0.01; ns, not significant by Dunnett’s test. ††p < 0.01 of the interaction effect analyzed by two-way ANOVA. Spearman correlation coefficient (r) was calculated and a test of no correlation was performed.

**Fig 3:**
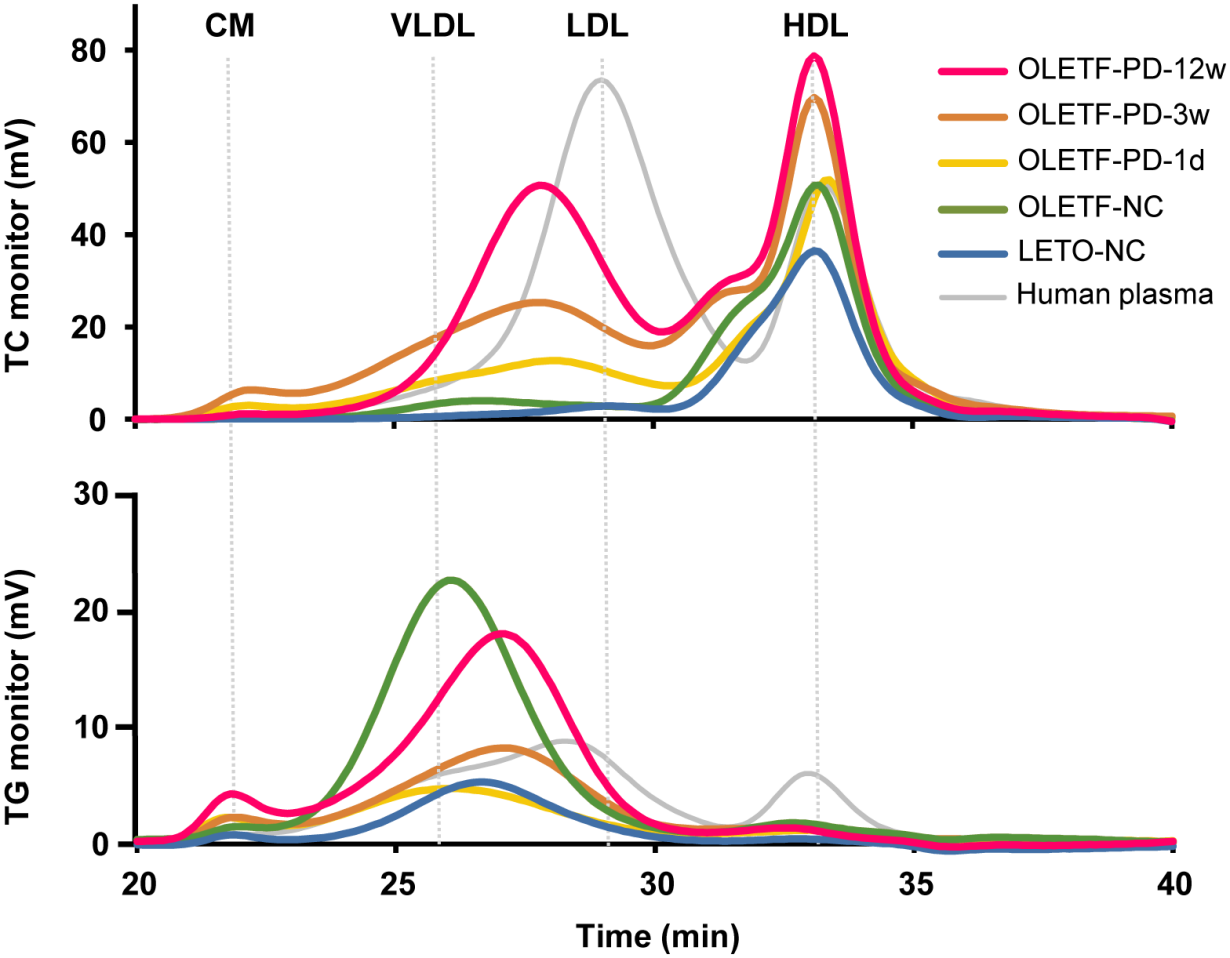
Plasma lipoprotein profiles in LETO and OLETF rats. Plasma lipoprotein profiles of rats aged 6 (OLETF-PD-1d), 9 (OLETF-PD-3w) and 18 weeks (LETO-NC, OLETF-NC and OLETF-PD-12w) were analyzed by HPLC using the pooled plasma of each group. CM, chylomicron; HDL, high-density lipoprotein; LDL, low-density lipoprotein; VLDL, very low-density lipoprotein.

Both the HF- and CA-diets tended to increase UAE and glomerular sclerosis in parallel with the increase in plasma TC concentration in OLETF rats, but PD increased both abnormalities far more than the HF or CA diets (Fig 2A–2C). Statistical analysis revealed that the effects of the HF- and CA-diets on the glomerular sclerosis index and plasma TC concentration are synergistic (Fig 2B and 2C). Changes in plasma TC concentrations were correlated well with changes in the UAE and glomerular sclerosis index in OLETF rats fed PD (Fig 2D), suggesting that increased plasma TC concentration is involved in the aggravation of kidney injury in OLETF rats. From the analysis of the size distributions of plasma lipoproteins by high performance liquid chromatography, we confirmed that the increased TC concentration with PD was mainly due to an increase in the LDL-VLDL fraction (Fig 3) as seen in other rodents and rabbits fed atherogenic diets [19–22]; suggesting that the fraction was responsible for the increased UAE.

The CA-diet increased kidney weight, plasma TG concentration, and excretion of urinary creatinine and 8-hydroxy-2'-deoxyguanosine, while the HF-diet had no effect on these parameters apart from a slight decrease in kidney weight (S2 Table).

### Glomerular and tubular injuries aggravated by the Paigen-diet

**Fig 4:**
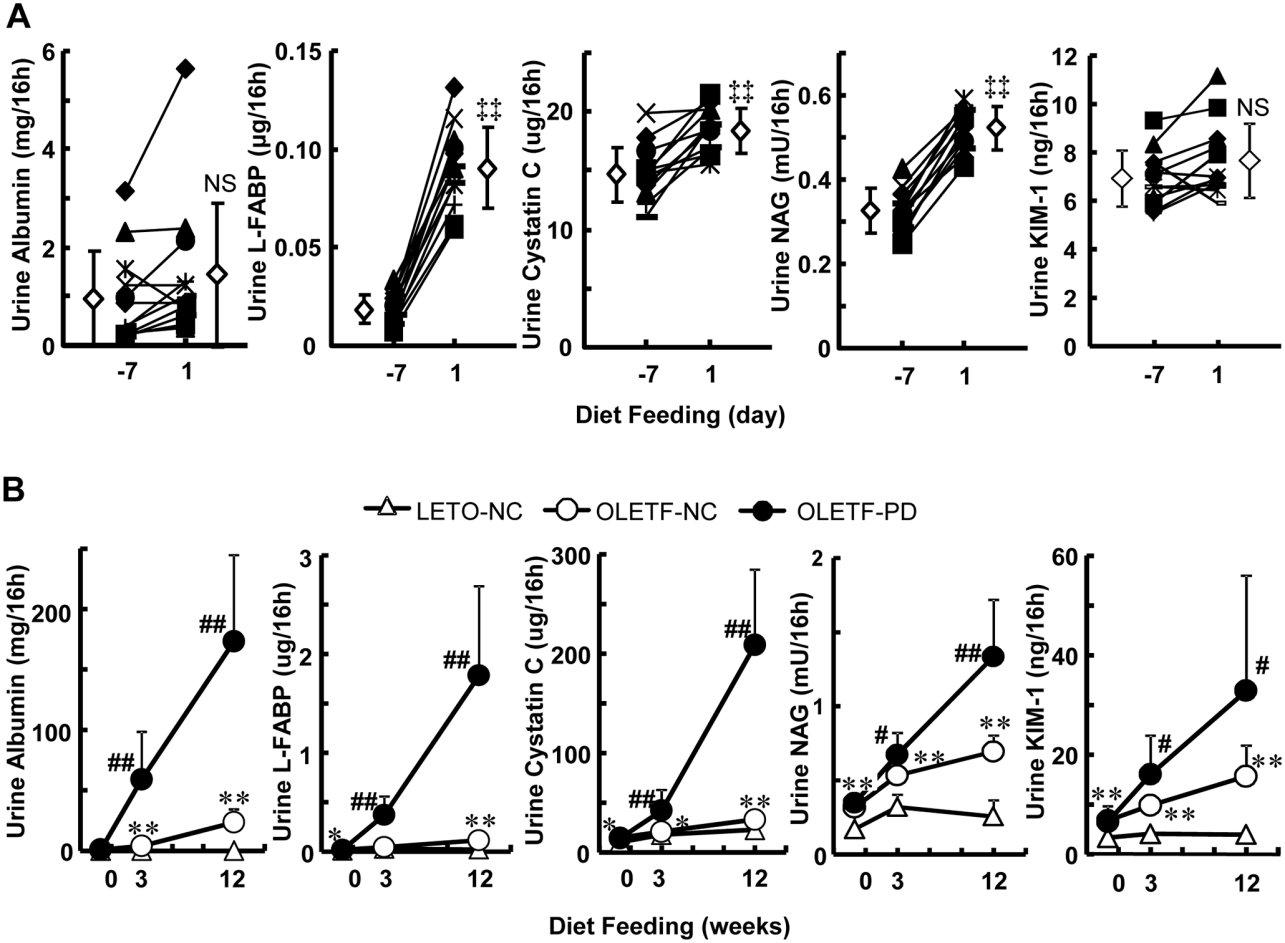
Time-dependent changes in urine excretion of tubule injury markers. Changes in urinary excretion of albumin, liver fatty-acid-binding protein (L-FABP), cystatin C, N-acetyl-β-D-glucosaminidase (NAG), and kidney injury molecule-1 (KIM-1) after starting to feed a Paigen diet in OLETF rats are presented as mean ± SD. (A) Changes between baseline and the day after starting to feed a Paigen diet in OLETF rats (n = 11). (B) Time-dependent changes from baseline to 12 weeks after the start of normal chow or Paigen diet feeding. There were five animals in the group of LETO rats fed normal chow (LETO-NC), eight in the group of OLETF rats fed normal chow (OLETF-NC), and 12 in the group of OLETF rats fed a Paigen diet (OLETF-PD). ‡‡p < 0.01; NS, not significant by paired t-test. *p < 0.05; **p < 0.01 vs LETO-NC group and #p < 0.05; ##p < 0.01 vs OLETF-NC group by unpaired t-test.

To examine tubular injury after PD feeding, we measured time-dependent changes in urinary excretion of tubular injury markers. Urinary excretion of identified biomarkers of acute kidney injury [23, 24], namely L-FABP, cystatin C and NAG, but not KIM-1, increased abruptly after the onset of PD feeding, and increments were significant on the following day, in advance of increments in UAE (Fig 4A). However, excretion increased further in a time-dependent manner (Fig 4B), and the extent of urinary excretion of the tubular injury paralleled UAE 12 weeks after PD feeding (S1 Fig). Plasma TC concentration (120 ± 11 mg/dL) before the start of PD feeding increased significantly the day after the start of PD feeding (186 ± 24 mg/dL) in OLETF rats, which is higher than that in LETO rats fed PD (Fig 1C). However, there was no positive correlation between plasma TC concentration and urinary excretion of tubular injury markers on that day (data not shown).

**Fig 5:**
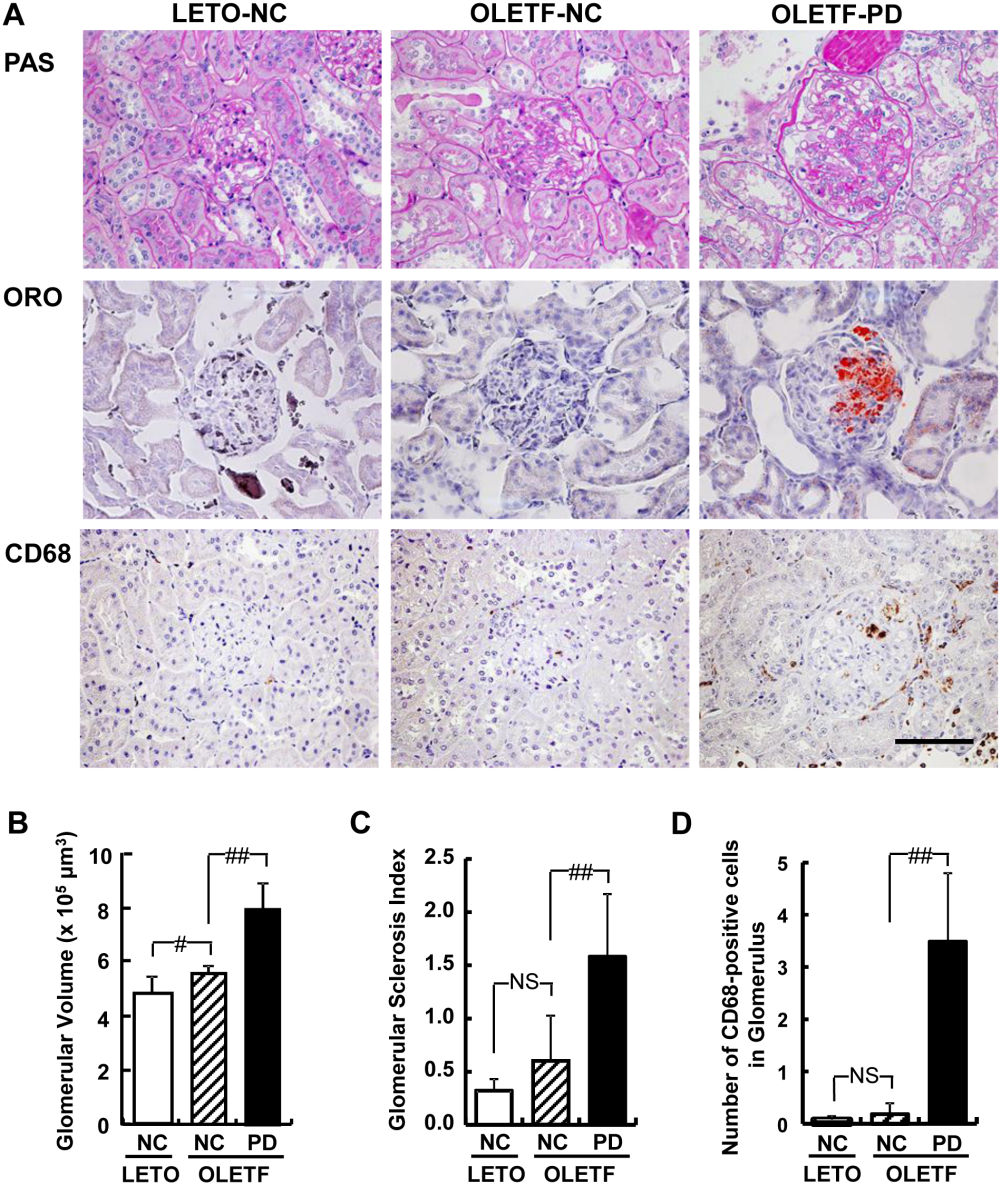
Histological analysis of kidneys in LETO and OLETF rats. (A) Representative histological images are shown of glomerular, tubular and tubulointerstitial lesions of each group 12 weeks after the start of Paigen diet feeding. Scale bar, 100 μm. Glomerular volume (B), glomerular sclerosis index (C) and macrophage (CD68)-positive cells in glomeruli (D) are presented as mean ± SD. There were five animals in the group of LETO rats fed normal chow (LETO-NC), eight in the group of OLETF rats fed normal chow (OLETF-NC), and 11 in the group of OLETF rats fed a Paigen diet (OLETF-PD). #p < 0.05; ##p < 0.01; NS, not significant by unpaired t-test. ORO, Oil red O stain; PAS, periodic acid schiff stain.

Histological analysis revealed that the glomerular lesion in OLETF rats fed PD was mainly composed of segmental mesangial proliferation, hyalinosis, sclerosis, capillary wall abnormalities, adhesions and crescent formation, and no nodular lesions were observed (Fig 5A). Glomerular volume in OLETF rats fed PD was significantly larger than that in OLETF rats fed NC (Fig 5A and 5B), and the expanded mesangial area induced by PD was reconfirmed in this experiment (Fig 5A and 5C). Thinning of the brush border, thickening of the basement membrane in the proximal tubule, and urinary casts were also observed in these rats (Fig 5A). Oil red O- and CD68-positive areas were observed in glomerular and proximal tubular lesions in OLETF rats fed PD (Fig 5A). CD68- positive cells were also observed in tubulointerstitial fields and the number of such cells in glomeruli increased significantly in OLETF rats fed PD (Fig 5A and 5D).

**Fig 6:**
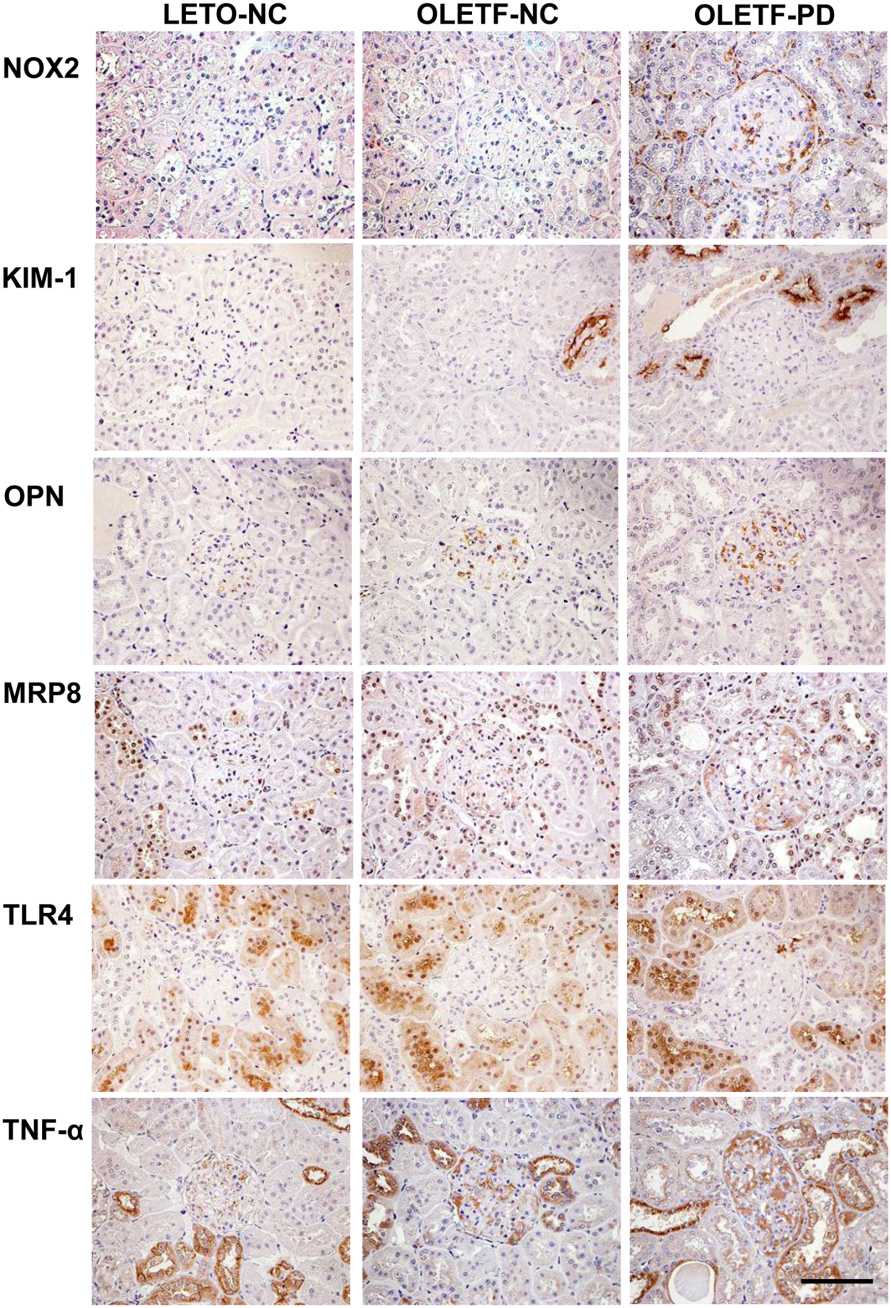
Immunohistochemical analysis of kidneys in LETO and OLETF rats. Representative immunohistochemical images of glomerular, tubular and tubulointerstitial lesions in each group, 12 weeks after starting to feed a Paigen diet. Scale bar, 100 μm. KIM-1, kidney injury molecule-1; MRP8, myeloid-related protein 8; NOX2, NADPH oxidase 2; OPN, osteopontin; TLR4, toll-like receptor 4; TNF-α, tumor necrosis factor-α.

The NOX2/Gp91phox-positive area increased in glomerular and tubulointerstitial fields in OLETF rats fed PD (Fig 6). In OLETF rats fed PD, we found intense KIM-1 and TLR4-positive areas in proximal tubules and intense MRP8- and TNF-α-positive areas in glomeruli and distal tubules (Fig 6). An osteopontin-positive area was observed in the glomerular endothelium in OLETF rats fed PD (Fig 6).

**Fig 7:**
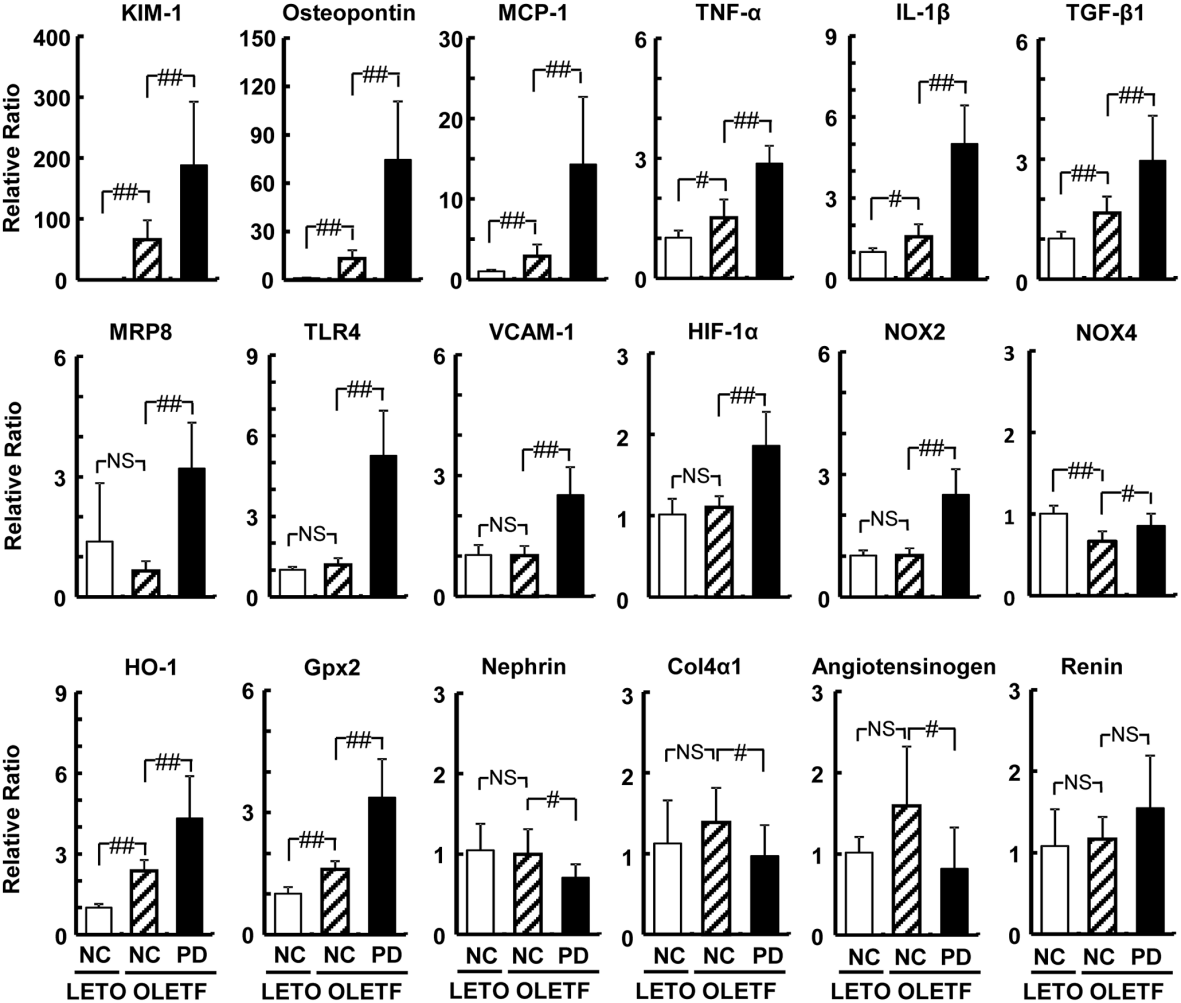
mRNA levels in kidneys of LETO and OLETF rats. The mRNA levels in kidneys of LETO and OLETF rats, 12 weeks after the start of Paigen diet feeding, are presented as mean ± SD. There were five animals in the group of LETO rats fed normal chow (LETO-NC), eight in the group of OLETF rats fed normal chow (OLETF-NC), and 12 in the group of OLETF rats fed a Paigen diet (OLETF-PD). #p < 0.05; ##p < 0.01; NS, not significant by unpaired t-test. Col4a1, procollagen type IV 4α; Gpx2, glutathione peroxidase 2; HIF-1α, hypoxia inducible factor 1α; HO-1, heme oxygenase 1; IL-1β, interleukin-1β; KIM-1, kidney injury molecule-1; MCP-1, monocyte chemoattractant protein-1; MRP8, myeloid-related protein 8; NOX2, NADPH oxidase 2; NOX4, NADPH oxidase 4; TGF-β, transforming growth factor-β; TLR4, toll-like receptor 4; TNF-α, tumor necrosis factor-α; VCAM-1, vascular cell adhesion molecule-1.

Analysis of renal mRNA expressions revealed that the mRNA levels related to inflammation and oxidative stress such as KIM-1, osteopontin, MCP-1, TNF-α, interleukin-1β, MRP8, TLR4, VCAM-1, hypoxia inducible factor 1α (Hif-1α), and NOX2, and anti-oxidative enzymes such as heme oxygenase 1 (HO-1) and glutathione peroxidase 2 (Gpx2) were significantly increased in OLETF rats fed PD (Fig 7). Expression of nephrin, a structural component of the slit diaphragm [25], significantly decreased in OLETF rats fed PD. Transforming growth factor β1 was upregulated but procollagen type IV 4α was not upregulated, and obvious fibrosis was not observed in the kidney in OLETF rats fed PD (data not shown). The levels of renin and angiotensinogen did not increase in OLETF rats fed PD.

Body and kidney weights and concentrations of plasma and urine parameters from the LETO-NC, OLETF-NC and OLETF-PD group were similar to those in Experiment 1 (S3 Table).

## Discussion

In this study, we clearly showed that kidney impairment in type 2 diabetic OLETF rats was abruptly worsened by the atherogenic PD. This deterioration was accompanied by severe glomerular and tubular injuries concomitant with massive urinary excretion of albumin and tubular injury markers. Intriguingly, PD caused negligible damage to the kidneys in normal LETO rats, suggesting that the genetic background of the OLETF rat strongly affects the vulnerability of its kidneys to this diet.

A major quantitative trait locus for dyslipidemia, obesity and a diabetic phenotype in OLETF rats is termed “diabetes mellitus OLETF type I” (*Dmo1*). Congenic rats, in which both OLETF *Dmo1* alleles are replaced by the genome derived from a normal strain F344, showed a significant decrease in food intake, body weight, abdominal fat weight, serum TC and TG, and blood glucose after glucose loading [26]. The congenic rats also showed less mesangial sclerosis and deposition of hyalin and PAS-positive substance in the kidney. These results suggest that *Dmo1* may be the locus responsible for the vulnerability of kidneys to PD in OLETF rats. The type 2 diabetes phenotype in OLETF rats is regulated by multiple genes [27], and the precise mechanism by which the phenotype develops is currently unknown. However, it is reported that OLETF rats lack the cholecystokinin-1 receptor; they are therefore obese [28]. Cholecystokinin-1 is a peptide hormone that stimulates postprandial satiety and digestion of fat and protein, and is reported to play a protective role in diabetic kidney disease by exerting anti-inflammatory actions on macrophages [29]; therefore, lack of the receptor may contribute to aggravation by PD.

OLETF rats already have some renal abnormalities in the prediabetic stage (at 4–13 week old) such as higher kidney angiotensin II contents and renal type IV collagen mRNA expression [30]; vascular narrowing and an impaired renal glomerular filtration capacity [31]; and abnormal autoregulation and tubuloglomerular feedback [32]. These phenotypes may also underlie the vulnerability of the kidneys to the diet.

PD did not change the plasma glucose concentration. However, it significantly increased plasma TC concentration, which correlates well with the increases in UAE and mesangial expansion, suggesting that increased plasma TC concentration aggravates kidney impairment. It was however reported that PD increased plasma TC concentration up to 6 000 mg/dL in streptozotocin-injected rats [18], but the increase in UAE (1.4 mg/day) was far less (< 100 mg/day) in OLETF rats fed PD. Moreover, kidney injury has not been reported in patients with familial hypercholesterolemia. Therefore, severe disease aggravation induced by a high plasma TC concentration would occur only in specific circumstances.

The mechanism by which increased plasma cholesterol worsens renal impairment in OLETF rats is currently unknown. It is reported that oxidized LDL upregulates TLR4 expression [33] which upregulates MCP-1 expression [34], and induces loss of nephrin expression from cultured podocytes [35]. Macrophage accumulation in glomerular and tubulointerstitial areas, induced by PD, would upregulate NOX2 expression, promote LDL oxidation and aggravate the nephropathy. In addition, positive correlations were observed between plasma TC concentration and renal expression of KIM-1 and osteopontin (S2 Fig). KIM-1 is a phosphatidylserine receptor expressed in damaged proximal tubular epithelial cells, associated with tubulointerstitial inflammation and fibrosis after ischemic kidney injury [23, 36]. It is reported that KIM-1 upregulates MCP-1 and induces kidney inflammation and fibrosis [37, 38], leading to further tubular damage and loss of renal function. On the other hand, osteopontin is a large phosphoglycoprotein adhesion molecule with pleiotropic effects that include promotion of macrophage accumulation. Osteopontin seems to be causally involved in the pathogenesis of diabetic nephropathy, as albuminuria and renal damage are ameliorated in osteopontin-deficient diabetic mice [39, 40]. Upregulation of these molecules after PD feeding may influence the disease aggravation that correlates with the increase in plasma cholesterol concentration.

Cholic acid not only promotes cholesterol absorption, but also enhances some inflammatory responses [41]. The early inflammatory response induced by PD is reported to be mediated partially through TLR4 [34]. On the other hand, MRP8, an endogenous activator of TLR4, increases the expression of TNF-α and IL-1β [42]. It is reported that MRP8 produced by infiltrating macrophages might exert glucolipotoxic effects upon diabetic glomeruli, potentially leading to mesangial expansion, podocyte injury, glomerular sclerosis and albuminuria [43, 44]. Moreover, kidney expression of MRP8 was significantly predictive in patients with obesity- or type 2 diabetes-associated kidney diseases [44]. Renal mRNA levels of MRP8 and TLR4 were increased by PD in this study, as reported previously in type 1 (streptozotocin-induced) and type 2 (A-ZIP/F-1 lipoatrophic) diabetic mice [43]. Therefore, MRP8 / TLR4 signaling may also contribute to the effect exerted by PD in the OLETF rat.

Increased oil-red O-positive areas in glomeruli and tubules induced by PD may partly be due to accumulation of fatty acids, as renal L-FABP expression was upregulated; however, plasma FFA concentration did not change. Increased urinary protein may promote tubulointerstitial damage [45], in part due to increased FFAs. FFAs bound to albumin are filtered through the glomeruli and reabsorbed into the proximal tubules. Therefore, in massive proteinuria, FFAs overload the proximal tubules and induce inflammatory factors such as macrophage chemotactic factors [46], with resulting tubulointerstitial damage [47]. Tubular epithelial cells, as the most abundant cell type in the renal cortex, play active roles not only in the progression of acute kidney injury but also in chronic kidney diseases. In this experiment, the increase in urinary excretion of tubular injury markers preceded the increase in UAE after PD feeding; however, the extent of the increases paralleled that of UAE 12 weeks after the start of PD feeding. It is suggested that tubular epithelial cells are more susceptible to PD than the other renal cells, but these cells may sustain more severe damage from FFAs once massive proteinuria has been induced. In this situation, increased urinary L-FABP may play a protective role against increased FFAs in urine because L-FABP has an anti-oxidative effect [48] and protects against renal injury [36].

The relationship between changes in the expression of molecules in the kidney and progression of kidney injury is very complex, because these molecules interact with one another and some injurious molecules also have protective effects on the kidney. For example, HIF-1, a hypoxia-inducible transcription factor, is postulated to play contrasting protective and pathogenic roles in acute and chronic kidney diseases, respectively [49]. Moreover, NOX2 expression was upregulated by PD, but the level of NOX4, a major NADPH oxidase in the kidney that is upregulated by high glucose [50], was not changed, and anti-oxidative enzymes such as HO-1 and Gpx2 were upregulated by PD in OLETF rats. These results suggest that regulation of the redox state is complex and this might be one reason why antioxidants per se have demonstrated minimal renoprotection in humans despite positive preclinical research findings. Further studies are needed to clarify the mechanism by which PD aggravates kidney impairment in OLETF rats.

OLETF rats have several renal abnormalities but without albuminuria in the prediabetic stage; suggesting that studying the relation between the preexisting abnormalities and kidney injuries after receiving a PD in association with tubulointerstitial lesions would be important in the endeavor to discover new methods of early diagnosis and treatment of diabetic kidney disease in patients without albuminuria as these patients represent a large percentage of those with the disease [51, 52]. In addition, there should be further study on the relationship between the developmental and pathological features of kidney injury in this animal model and those in humans with dyslipidemia, such as refractory nephrotic syndrome, lipoprotein glomerulopathy and familial lecithin cholesterol acyltransferase deficiency.

In conclusion, aggravation of DN by an atherogenic diet was strongly affected by genetic background; increased plasma cholesterol seems to play an important role. The pathogenesis of DN is multi-factorial, and a multi-pronged drug approach that targets blood pressure and serum levels of glucose, insulin, cholesterol and TG fails to fully prevent DN. This model would be useful to study the complex mechanisms of initiation and progression of kidney injury that occur in an atherogenic environment, and in developing new methods for early diagnosis and treatment of diabetic kidney disease.

## Supporting Information

S1 Fig Relationships between urinary excretions of albumin and kidney injury markers. Relationships are shown between urinary excretion of albumin and kidney injury markers 12 weeks after PD feeding. The Pearson or Spearman correlation coefficient (r) was calculated. KIM-1, kidney injury molecule-1; L-FABP, liver fatty-acid-binding protein; NAG, N-acetyl-β-D-glucosaminidase.
(TIF)

S2 Fig Relationships between plasma total cholesterol concentration and various mRNA levels in kidney. Relationships are shown between plasma total cholesterol concentration (TC) and various mRNA levels in renal tissue the day after the start of PD feeding. The Pearson correlation coefficient(r) was calculated and a test of no correlation was performed. HIF-1α, hypoxia inducible factor 1α; IL-1β, interleukin-1β; KIM-1, kidney injury molecule-1; MCP-1, monocyte chemoattractant protein-1; MRP8, myeloid-related protein 8; NOX2, NADPH oxidase 2; TGF-β, transforming growth factor β; TLR4, toll-like receptor 4; TNF-α, tumor necrosis factor α; VCAM-1, vascular cell adhesion molecule 1.
(TIF)

S1 Table Primer information. (DOC)

S2 Table Metabolic parameters of OLETF rats, 5 weeks after being fed each diet in Experiment 2. Values obtained 5 weeks after feeding on each diet are presented as mean ± SD. *p<0.05, **p<0.01 compared to the OLETF-NC group by Dunnett’s test. Other comparisons with the OLETF-NC group were not significant (Dunnett’s test). BW, body weight; CA, 0.5% sodium cholate-supplemented diet; CCr, creatinine clearance; FI, food intake; HF, high-fat diet; KW, kidney weight; NC, normal chow; pCre, plasma creatinine concentration; pGlu, plasma glucose concentration; pIns, plasma insulin concentration; pTG, plasma triglyceride concentration; uCre, urine creatinine excretion; u8OHdG, urine 8-hydroxydeoxyguanosine excretion; UV, urine volume; WI, water intake.
(DOC)

S3 Table Metabolic parameters of LETO and OLETF rats, 12 weeks after being fed chow or PD in Experiment 3. Values obtained 12 weeks after feeding on each diet are presented as mean ± SD. #p < 0.05, ##p < 0.01 compared to the OLETF-NC group by unpaired t-test. Other comparisons with the OLETF-NC group were not significant (unpaired t-test). BW, body weight; KW, kidney weight; FI, food intake; WI, water intake; pGlu, plasma glucose concentration; pIns, plasma insulin concentration; pTC, plasma total cholesterol concentration; pTG, plasma triglyceride concentration; pNEFA, plasma non-esterified fatty acid concentration; pUN, plasma urea nitrogen concentration; pCre, plasma creatinine concentration; CCr, creatinine clearance; UV, urine volume; uCre, urine creatinine excretion; u8OHdG, urine 8- hydroxydeoxyguanosine excretion; NC, normal chow.
(DOC)
