# Supplementary figures and images for: An Atherogenic Paigen-Diet Aggravates Nephropathy in Type 2 Diabetic OLETF Rats
Source: PLoS One. 2015 Nov 25;10(11):e0143979. doi: 10.1371/journal.pone.0143979 (PMC4659596; doi:10.1371/journal.pone.0143979)

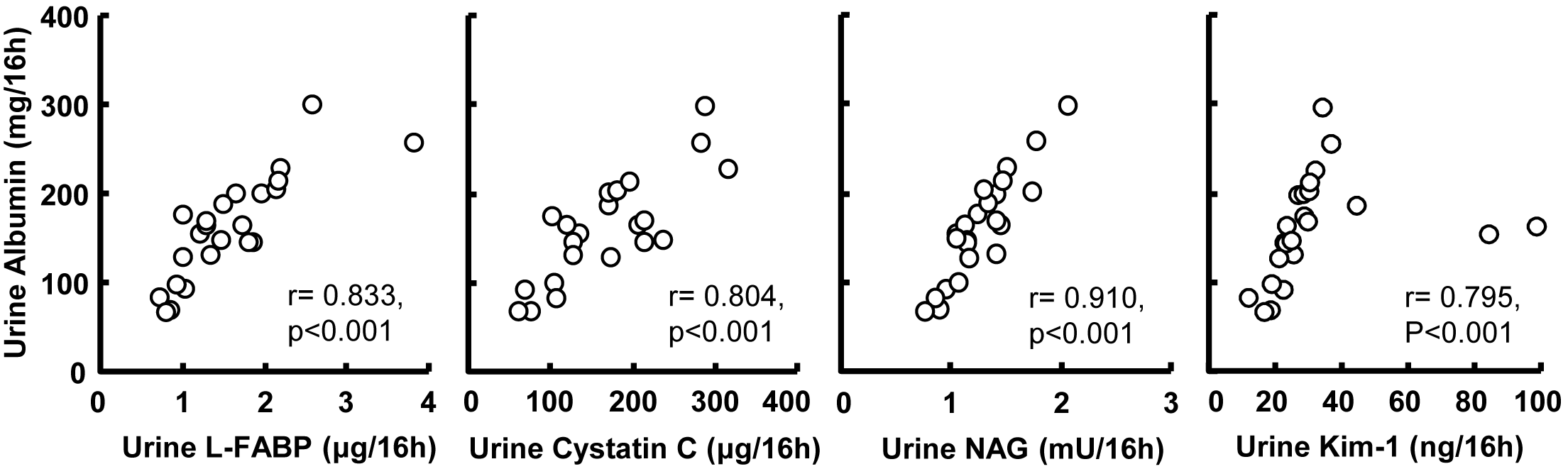

Supplement: S1 Fig — Relationships are shown between urinary excretion of albumin and kidney injury markers 12 weeks after PD feeding. The Pearson or Spearman correlation coefficient (r) was calculated. KIM-1, kidney injury molecule-1; L-FABP, liver fatty-acid-binding protein; NAG, N-acetyl-β-D-glucosaminidase. (TIF) [file pone.0143979.s001.tif]

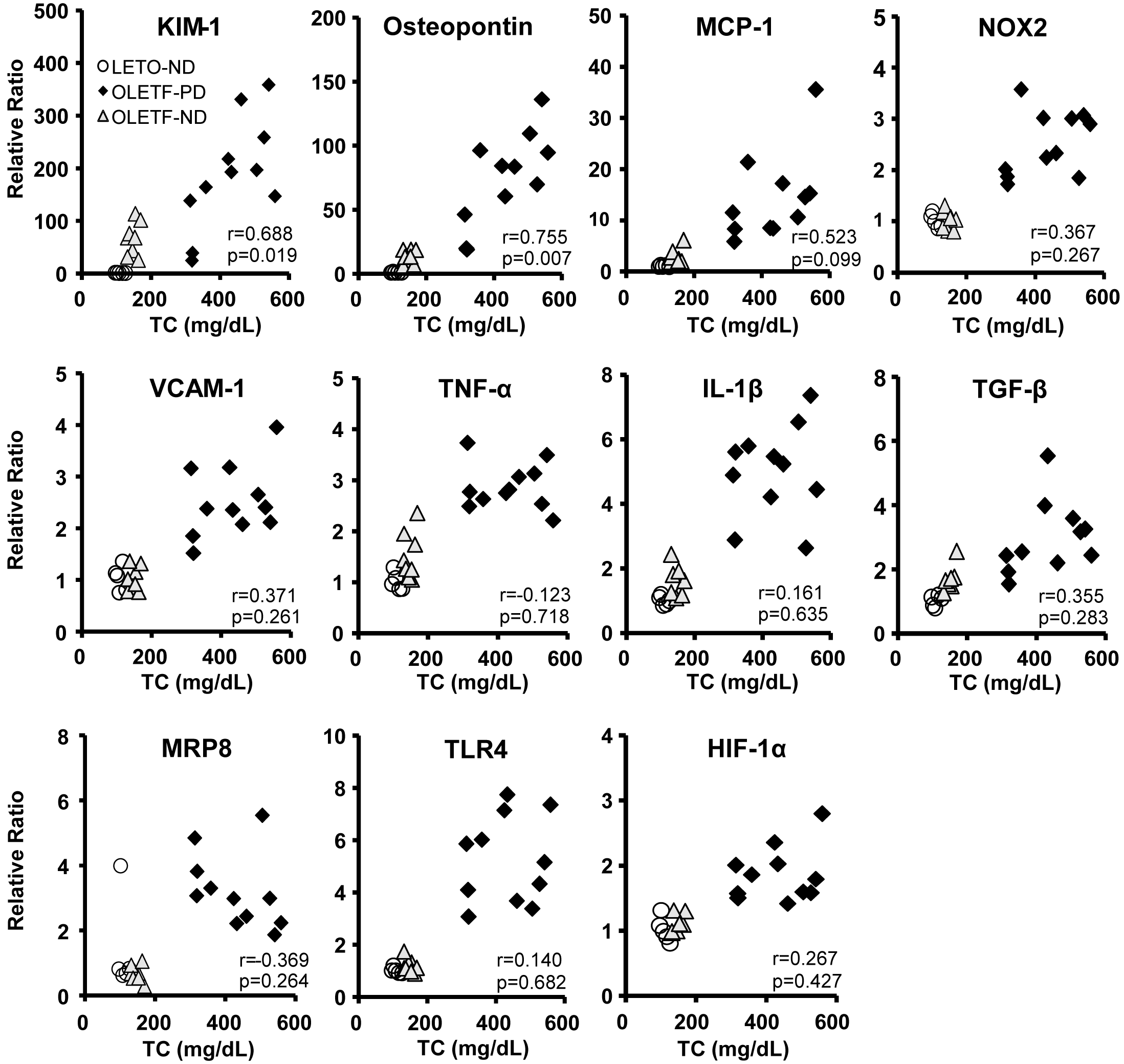

Supplement: S2 Fig — Relationships are shown between plasma total cholesterol concentration (TC) and various mRNA levels in renal tissue the day after the start of PD feeding. The Pearson correlation coefficient(r) was calculated and a test of no correlation was performed. HIF-1α, hypoxia inducible factor 1α; IL-1β, interleukin-1β; KIM-1, kidney injury molecule-1; MCP-1, monocyte chemoattractant protein-1; MRP8, myeloid-related protein 8; NOX2, NADPH oxidase 2; TGF-β, transforming growth factor β; TLR4, toll-like receptor 4; TNF-α, tumor necrosis factor α; VCAM-1, vascular cell adhesion molecule 1. (TIF) [file pone.0143979.s002.tif]
