# Supplementary material for: An Atherogenic Paigen-Diet Aggravates Nephropathy in Type 2 Diabetic OLETF Rats
Source: PLoS One. 2015 Nov 25;10(11):e0143979. doi: 10.1371/journal.pone.0143979 (PMC4659596; doi:10.1371/journal.pone.0143979)
Supplement: S1 Table — (DOC) [file pone.0143979.s003.doc]

| Gene | Product name | Catalogue No. |
| --- | --- | --- |
| Havcr1 (KIM-1) | Rn_Havcr1_1_SG QuantiTect Primer Assay | QT00185983 |
| Spp1 (Osteopontin) | Rn_Spp1_1_SG QuantiTect Primer Assay | QT00199101 |
| Ccl2 (MCP-1) | Rn_Ccl2_1_SG QuantiTect Primer Assay | QT00183253 |
| Tnf (TNF-α) | Rn_Tnf_1_SG QuantiTect Primer Assay | QT00178717 |
| Il1b (IL-1β) | Rn_Il1b_1_SG QuantiTect Primer Assay | QT00181657 |
| Tgfb1 (TGF-β1) | Rn_Tgfb1_3_SG QuantiTect Primer Assay | QT02488178 |
| S100a8 (MRP8) | Rn_S100a8_1_SG QuantiTect Primer Assay | QT00402969 |
| Tlr4 (TLR4) | Rn_Tlr4_1_SG QuantiTect Primer Assay | QT00387184 |
| Vcam1 (VCAM-1) | Rn_Vcam1_1_SG QuantiTect Primer Assay | QT00178500 |
| Hif1a (HIF-1α) | Rn_Hif1a_1_SG QuantiTect Primer Assay | QT00182532 |
| Cybb (NOX2) | Rn_RGD:620574_1_SG QuantiTect Primer Assay | QT00195300 |
| Nox4 (NOX4) | Rn_Nox4_1_SG QuantiTect Primer Assay | QT00186550 |
| Hmox1 (HO-1) | Rn_Hmox1_1_SG QuantiTect Primer Assay | QT00175994 |
| Gpx2 | Rn_Gpx2_1_SG QuantiTect Primer Assay | QT00375501 |
| Nphs1 (Nephrin) | Rn_Nphs1_1_SG QuantiTect Primer Assay | QT00189805 |
| Col4a1 | Rn_Col4a1_1_SG QuantiTect Primer Assay | QT01620073 |
| Agt (Angiotensinogen) | Rn_Agt_1_SG QuantiTect Primer Assay | QT00177478 |
| Ren (Renin) | Rn_Ren_1_SG QuantiTect Primer Assay | QT00183435 |
| Gapd (GAPDH) | Rn_Gapd_1_SG QuantiTect Primer Assay | QT00199633 |
