# Supplementary material for: An Atherogenic Paigen-Diet Aggravates Nephropathy in Type 2 Diabetic OLETF Rats
Source: PLoS One. 2015 Nov 25;10(11):e0143979. doi: 10.1371/journal.pone.0143979 (PMC4659596; doi:10.1371/journal.pone.0143979)
Supplement: S2 Table — Values obtained 5 weeks after feeding on each diet are presented as mean ± SD. *p<0.05, **p<0.01 compared to the OLETF-NC group by Dunnett’s test. Other comparisons with the OLETF-NC group were not significant (Dunnett’s test). BW, body weight; CA, 0.5% sodium cholate-supplemented diet; CCr, creatinine clearance; FI, food intake; HF, high-fat diet; KW, kidney weight; NC, normal chow; pCre, plasma creatinine concentration; pGlu, plasma glucose concentration; pIns, plasma insulin concentration; pTG, plasma triglyceride concentration; uCre, urine creatinine excretion; u8OHdG, urine 8-hydroxydeoxyguanosine excretion; UV, urine volume; WI, water intake. (DOC) [file pone.0143979.s004.doc]

|  | OLETF  -NC | OLETF  -HF | OLETF  -CA | OLETF  -Paigen |
| --- | --- | --- | --- | --- |
| BW (g) | 479 ± 26 | 472 ± 25 | 476 ± 21 | 497 ± 30 |
| KW (g) | 2.8 ± 0.1 | 2.6 ± 0.1** | 3.0 ± 0.1* | 2.9 ± 0.2 |
| FI (g/day) | 31 ± 2 | 27 ± 1 | 31 ± 2 | 28 ± 2* |
| WI (g/day) | 49 ± 6 | 45 ± 6 | 51 ± 9 | 51 ± 9 |
| pGlu (mg/dL) | 160 ± 11 | 170 ± 12 | 163 ± 13 | 168 ± 14 |
| pIns (ng/mL) | 4.1 ± 1.7 | 4.0 ± 1.2 | 2.9 ± 0.9 | 4.9 ± 3.2 |
| pTG (mg/dL) | 150 ± 34 | 186 ± 72 | 300 ± 50* | 196 ± 122 |
| pCre (mg/dL) | 0.62 ± 0.04 | 0.66 ± 0.04 | 0.65 ± 0.08 | 0.69 ± 0.06 |
| CCr (mL/min) | 1.91 ± 0.29 | 1.72 ± 0.35 | 2.14 ± 0.37 | 1.82 ± 0.29 |
| uCre (mg/16 h) | 11.3 ± 1.7 | 10.8 ± 1.9 | 13.2 ± 1.3* | 11.8 ± 1.1 |
| u8OHdG (ng/16 h) | 268 ± 124 | 213 ± 80 | 428 ± 115* | 363 ± 54 |
| UV (mL/16 h) | 17 ± 5 | 19 ± 7 | 15 ± 7 | 15 ± 7 |
| n | 8 | 8 | 8 | 8 |
