# Supplementary material for: An Atherogenic Paigen-Diet Aggravates Nephropathy in Type 2 Diabetic OLETF Rats
Source: PLoS One. 2015 Nov 25;10(11):e0143979. doi: 10.1371/journal.pone.0143979 (PMC4659596; doi:10.1371/journal.pone.0143979)
Supplement: S3 Table — Values obtained 12 weeks after feeding on each diet are presented as mean ± SD. #p < 0.05, ##p < 0.01 compared to the OLETF-NC group by unpaired t-test. Other comparisons with the OLETF-NC group were not significant (unpaired t-test). BW, body weight; KW, kidney weight; FI, food intake; WI, water intake; pGlu, plasma glucose concentration; pIns, plasma insulin concentration; pTC, plasma total cholesterol concentration; pTG, plasma triglyceride concentration; pNEFA, plasma non-esterified fatty acid concentration; pUN, plasma urea nitrogen concentration; pCre, plasma creatinine concentration; CCr, creatinine clearance; UV, urine volume; uCre, urine creatinine excretion; u8OHdG, urine 8- hydroxydeoxyguanosine excretion; NC, normal chow. (DOC) [file pone.0143979.s005.doc]

|  | LETO  -NC | OLETF  -NC | OLETF  -Paigen |
| --- | --- | --- | --- |
| BW (g) | 479 ± 36## | 670 ± 27 | 654 ± 31 |
| KW (g) | 2.6 ± 0.1## | 4.0 ± 0.4 | 4.0 ± 0.5 |
| FI (g/day) | 24 ± 1## | 33 ± 2 | 33 ± 2 |
| WI (g/day) | 37 ± 5## | 45 ± 3 | 54 ± 6## |
| pGlu (mg/dL) | 138 ± 7## | 171 ± 16 | 169 ± 36 |
| pIns (ng/mL) | 2.6 ± 0.5## | 12.6 ± 3.8 | 5.9 ± 1.5## |
| pTC (mg/dL) | 110 ± 12## | 149 ± 14 | 433 ± 94## |
| pTG (mg/dL) | 67 ± 24## | 303 ± 108 | 295 ± 133 |
| pNEFA (mEq/L) | 0.60 ± 0.34 | 0.39 ± 0.09 | 0.86 ± 0.17## |
| pUN (mg/dL) | 26 ± 3 | 29 ± 8 | 20 ± 4## |
| pCre (mg/dL) | 0.61 ± 0.10# | 0.89 ± 0.26 | 1.85 ± 0.84## |
| CCr (mL/min) | 1.98 ± 0.33 | 1.56 ± 0.68 | 0.91 ± 0.33# |
| uCre (mg/16 h) | 11.4 ± 0.6 | 12.2 ± 1.8 | 13.8 ± 1.4# |
| u8OHdG (ng/16 h) | 99 ± 65# | 180 ± 60 | 161 ± 35 |
| UV (mL/16 h) | 20 ± 6# | 12 ± 2 | 14 ± 3 |
| n | 5 | 8 | 11 |
